# Does Digital Capability Promote Sustainable Development of New Ventures? The Dual Impact of Green Knowledge Creation and Green Pressure

**DOI:** 10.3390/ijerph20032274

**Published:** 2023-01-27

**Authors:** Kai Zhuge, Weiwei Lin, Yongzhi Yuan, Huitao He, Yong Zhang

**Affiliations:** 1 Business School, Soochow University, Suzhou 215021, China; 2 School of Political and Public Administration, Soochow University, Suzhou 215021, China; 3 Institute of Quality Economics, China Jiliang University, Hangzhou 310018, China

**Keywords:** digital capability, green knowledge creation, green pressure, sustainable development, new ventures

## Abstract

With the environmental problems brought about by the extensive economic development model attracting more and more global attention, sustainable development has become a hot topic in transformation and development of contemporary enterprises. In the context of the digital economy, there is a lack of conclusive evidence regarding whether and how enterprises rely on digital capabilities to improve green efficiency and achieve sustainable development, especially for new ventures. Therefore, based on the knowledge creation spiral theory, this paper examines the relationship between digital capabilities, green knowledge creation, and sustainable development of new ventures. The study tested its hypotheses on a sample of 316 Chinese new ventures. The results showed that digital capabilities can positively affect environmental, economic, and social performance of new ventures and support their sustainable development; green knowledge creation plays a mediating role in the relationship between digital capabilities and sustainable development, and green pressure positively moderates the relationship between green knowledge creation and sustainable development; furthermore, green pressure also moderates the process of digital capabilities influencing sustainable development through green knowledge creation, and that moderated mediation role is significant. According to the bootstrap mediating effect test, both the direct effect and indirect effect are significant. Overall, our research results provide important insights for new ventures to promote sustainable development through digitalization. Therefore, managers should pay more attention to digital construction in the strategic layout of new ventures, and they should advocate the concept of green knowledge so that the goal of sustainable development can be achieved with the drive for digitalization.

## 1. Introduction

With the frequent occurrence of ‘black swan’ events, such as global warming, energy crises, major public health and security crises, and ecological and environmental problems brought about by the resource-led economic development model, achievement of sustainable development goals is facing major challenges. In this context, in November 2022, the 27th Climate Change Conference of the United Nations called for working ‘Together for Implementation’ of the green commitment, increasing climate financing and promoting green and sustainable development. Likewise, the ‘Guiding Opinions on Strengthening Industry–Finance Cooperation to Promote Industrial Green Development’, jointly released in 2021 by the Ministry of Industry and Information Technology and another four government departments in China, proposed expanding digital green consumption scenarios to support greenness with intelligence and establish commercially sustainable industry–finance cooperation to promote green development. The sustainability of the economy has become the focus of market ecological constructs [1]. Implementation of sustainable development strategies not only helps to build a resource-intensive and ecologically friendly market environment but is also a necessary choice for enterprises to establish competitive advantages and achieve sustainable development.

Meanwhile, with the advent of Industry 4.0, the iterations and changes of new generation digital technologies, such as big data, cloud computing, blockchain, and artificial intelligence, have promoted development of the digital economy [2]. Digital transformation is gradually becoming the core of corporate strategic changes. Digital capabilities are a dynamic attribute whereby enterprises introduce digital technologies into their existing management system to promote reshaping of corporate products and the value of services [3]. As the power foundation and essential conditions of enterprise transformation and upgrading, digital capabilities play a key role in improving organizational efficiency, reducing operating costs, and establishing a competitive advantage [4,5]. Studies have shown that, in the digital economy era, enterprises can improve their digital capabilities as an empowerment mechanism. From the perspective of resources, Khin and Ho (2019) believe that digital technology resources have provided enterprises broader potential for innovation, and enterprises can establish commercial sustainability based on a digital orientation [6]. From the perspective of dynamic ability, Ansong and Boineng (2019) believe that digital capabilities provide strategic tool support for interpretation and evaluation of big data resources of enterprises, thereby improving competitive advantages and output capabilities [7]. Digital empowerment makes value creation of enterprises no longer irregular, and improvement in visualization and traceability enables enterprises to effectively improve the efficiency, operating costs, and risk prevention and control of the company, thereby achieving the goal of sustainable development.

However, does digital capability really promote the sustainable development of an enterprise? In recent years, the relationship between digital capabilities and corporate development has received widespread academic attention [8], but the literature still has several shortcomings. First, many researchers have discussed digital capability from the perspective of business value, such as the relationship of digital capability with knowledge structure [9], organizational strategy [10], and business model innovation [11], but they have paid less attention to the impact of enterprises’ implementation of digitalization on environmental performance, green output, and other sustainability indicators. Second, knowledge is the most critical strategic resource for enterprises, and sufficient knowledge resources can enhance the competitive advantages of an enterprise [12]. For new ventures, digital capabilities can stimulate enterprises to absorb and reshape green knowledge, enable enterprises to establish green knowledge barriers, and maintain sustainable competitiveness; however, studies have rarely explored digitalization and sustainable development of enterprises from the perspective of knowledge creation. Finally, green pressure reflects an enterprise’s comprehensive perception of environmental awareness and behavior of stakeholders [13,14], and green pressure can be an important contingency factor in the process of creating green knowledge to achieve sustainable development. However, the extant literature on green pressure is limited to effects of supervision and managers, neglecting environmental requirements of stakeholders. In fact, the role of stakeholders in promoting green industry is crucial because their pursuit of environmental value is an important part of motivating an enterprise to implement a green strategy. In summary, there seems to be a theoretical gap in explaining how digital capabilities can build a bridge between green knowledge creation and green pressure for sustainable development. This paper aims to explore whether the digital capabilities of new ventures under green pressure can affect sustainable development through green knowledge creation.

Therefore, our research team designed this study to determine whether digital capabilities affect sustainable development of new ventures. A questionnaire-based survey was conducted to collect empirical data from 316 new ventures in China. Least squares regression was then used as our estimation method to test the hypotheses. The main conclusions were as follows: digital capabilities can positively affect sustainable development of new ventures; green knowledge creation plays a mediation role in the relationship between digital capabilities and sustainable development of new ventures; and green pressure positively moderates the relationship between green knowledge creation and sustainable development of new ventures. Furthermore, green pressure plays a significant moderating role in the process of digital capability influencing sustainable development through green knowledge creation; that is, the moderated mediation role is significant.

Our research has supplemented and expanded knowledge regarding the impact of digitalization on sustainable development in the following ways. First, based on knowledge creation screw theory, we proposed the theoretical model of ‘digital capability–green knowledge creation–sustainable development’ from the perspectives of knowledge socialization, externalization, combination, and internalization, explaining the process of creating green knowledge and its driving role. Second, this study has further enriched decisive factor research on sustainable development of enterprises. Since Elkington (1998) proposed the first three-line principle of sustainable development (environmental bottom line, economic bottom line, and social bottom line) [15], academic circles have discussed the impact on sustainable development and innovation from multiple perspectives, such as stakeholders [16], organizational structure [17], and absorptive capacity [18]. However, using the perspective of digital capability, we provide a new interpretation for sustainable development. Finally, this study incorporated the impact of green pressure on green knowledge creation. Du et al. (2018), based on the theory of resource dependence, proved that pressure of green stakeholders has a positive impact on green innovation by enterprises [19], showing that green behavior of enterprises is influenced by external pressure and that this impact is often positive.

The differences between our research and the existing research are as follows: (1) we focus on the impact of changes in digital capabilities, which is a critical but poorly researched area among the influencing factors of sustainable development. (2) Our analysis is based on the situation for new ventures in China and aims to provide decision-making solutions for sustainable development of such new ventures. (3) We also discussed the potential driving effect of different digital capabilities on green knowledge creation of enterprises and the regulatory role of green pressure faced by enterprises. In general, our research has filled several research gaps and provided new insights to help enterprises implement sustainable development strategies.

The rest of our paper is organized as follows. In Section 2, we provide a literature review and the theoretical assumptions. In Section 3, we discuss the data sources and variable measurements. In Section 4, we present the results of the reliability and validity test, the common method deviation test, and the hypothesis test. Finally, in Section 5, we present the conclusions, management implications, and future prospects of this research.

## 2. Theoretical Background and Hypothesis Development

### 2.1. The Impact of Digital Capability on Sustainable Development of New Ventures

Sustainable development of enterprises means that, in the process of survival and development, enterprises should not only pursue realization of short-term economic goals but also pay attention to material and energy trade-offs between the enterprises and the environment to maintain sustainable competitive advantages and achieve sustainable development [20]. Sustainable development of enterprises requires managers to establish long-term strategic change thinking rather than simply following previous business practices and only focusing on financial performance or commercial achievements. Especially for new ventures, Hockerts and Wustenhagen (2010) believe that it was new entrepreneurs who are more likely to pursue sustainable development opportunities while incumbent enterprises will merely respond to the actions of new entrants in adopting sustainable development practices [21]. Divito and Bohnsack (2017) pointed out that, with respect to sustainable decision-making, entrepreneurs will show more initiative and risk-taking in the direction of sustainable entrepreneurship [22]. As they are seldom constrained by organizational conventions, the sustainability motivations of new ventures are more often derived from internal motivations and the knowledge reserve of individual entrepreneurs [23].

In the context of digital transformation, sustainable development has become an important theme in the strategic agendas of enterprises. According to the forecast of the International Energy Agency, application of digital technology will reduce production costs of oil and gas by 10 to 20 percent, thereby eliminating 30 million tons of carbon dioxide emissions by 2040 [24]. Enterprises need to effectively balance digital tools to ensure intelligent applications and green characteristics [25] and enhance the impact of digital resources and capabilities for sustainability of enterprises through wise decisions [26]. Hagstrom (2012) believe that application capabilities of digital technologies, such as big data, represent a new paradigm for intellectual assets, symbolizing expansion and upgrading of enterprises’ intangible assets, making competitive advantages of enterprises no longer imitable [27]. Kunkel and Mathes (2020) proposed that, with development of information and communication technology capabilities, their impact on environmental sustainability has become more obvious [28]. Digital transformation represents an improvement in efficiency, requires energy-consuming enterprises to implement new production methods, improves the accuracy of process management, and enables enterprises to effectively achieve energy conservation and emission reduction while improving productivity. For example, the proposal of a ‘smart heating’ solution, using technologies such as big data analysis and algorithm modeling capabilities as the underlying framework, enabled Harbin Municipal Heating Corporation to effectively alleviate the problems of uneven heating, excessive energy consumption, and excessive emissions of polluting gas [29].

For new ventures with a lack of entrepreneurial resources, digital capabilities can help them better establish a ‘low-energy-consumption’ entrepreneurial concept [30] while effectively adapting to turbulence of changes in the environment; digital capabilities can also shorten response time to external environmental changes and accelerate the decision-making process, thereby ensuring continuity of business operations and green supply and realizing sustainability of environmental and economic performance [31]. Therefore, to achieve sustainable development, new ventures need to cultivate digital capability to improve entrepreneurial efficiency and their endowment of innovative resources. First, enterprises with high digital capabilities can achieve economic benefits by reducing operating costs and enhancing competitiveness while also avoiding opportunist behavior and failure risks in an uncertain economic environment. Second, digital capabilities can help enterprises to better monitor their environmental performance in the survival and growth stages and improve their green perception ability and the quality of their environmental information disclosure. Based on the above analysis, this paper proposes the following hypothesis.

**Hypothesis** **1** **(H1).** *Digital capability positively affects sustainable development of new ventures*.

### 2.2. The Impact of Digital Capability on Green Knowledge Creation

According to the theory of knowledge creation spiral, enterprises are entities committed to knowledge creation. Enterprises maintain their competitive advantage by acquiring, retaining, integrating and creating knowledge, and enriched knowledge resources can improve the sustainability of enterprise competition [32]. Spiral creation of knowledge is composed of interacting behaviors connected with tacit knowledge and explicit knowledge, including four processes: socialization, externalization, combination, and internalization [33]. Socialization refers to the process of transforming individual tacit knowledge into new tacit knowledge through sharing of experience and knowledge in the organization. Externalization refers to the process of translating tacit knowledge in the organization into a form of knowledge that is easy to be understood by and disseminated to the outside world. The combination process is for transforming the explicit knowledge mastered by the organization into a systematic knowledge system through innovation. The internalization process represents transformation from explicit knowledge to tacit knowledge. The socialization, externalization, combination, and internalization processes of knowledge form the behavior foundation of corporate knowledge creation. Liu and Chen (2017) believe that green knowledge is an important resource for enterprises to solve environmental problems by developing products and process innovation [34]. Zhang et al. (2021) believes that new green knowledge can not only accelerate the organizational learning process of enterprises but also help to implement the concept of green development into the actual production, operation, marketing, and service processes, thus helping enterprises to quickly form competitive advantages [35]. Sustainable development of new ventures needs to rely on rich green knowledge resources to translate and share discrete green information in the enterprise through the dynamic knowledge creation process and to promote green regeneration of knowledge.

Digital transformation of enterprises can largely eliminate the barriers to green information transmission, help in acquisition of remote green knowledge at a lower cost, and promote creation and spillover of knowledge. At the same time, digital capabilities can also promote exploration of new knowledge and technologies and help in acquiring knowledge, processing knowledge, and using knowledge in a more efficient way [36,37,38]. Green knowledge creation is the ability of enterprises to use all available resources, including technical resources, to identify, organize, and develop green knowledge to promote an enterprise’s continuous adaptation in demanding and even creating market changes. The driving role of digital capabilities in green knowledge creation is specifically reflected in three aspects: first, digital capabilities accelerate the green knowledge generated by green R&D cooperation between enterprises. Green R&D enables enterprises to exchange ideas and share knowledge on low-carbon issues, and digital capabilities strengthen the green knowledge connection between enterprises [39,40]; for example, big data analysis can further clarify the green needs of clients, and establishment of digital platforms deepens the green knowledge collaboration among enterprises. Second, digital capabilities affect knowledge creation by improving green learning efficiency. Generally, new ventures can acquire new technical knowledge through exploitative learning and exploratory learning. Digital transformation can greatly extend the boundary of entrepreneurial learning [41] and facilitate green production collaboration, supply collaboration, and marketing collaboration in cross-organization, cross-region, and cross-industry learning networks to accelerate green knowledge creation. Finally, application of digital technology improves employees’ digital literacy and enables them to rely on digital tools to accurately identify and judge green information [42], which is conducive to improving resource utilization and green R&D efficiency. Based on the above analysis, this paper proposes the following hypothesis.

**Hypothesis** **2** **(H2).** *Digital capability positively affects green knowledge creation*.

### 2.3. The Mediating Effect of Green Knowledge Creation

In the era of digitalization, enterprises not only need to use digital transformation to improve organizational operation efficiency but also need to fully use digital technology to explore new knowledge points and promote the transformation of knowledge from ‘realizing value’ to ‘creating value’ [43]. Czarnitzki and Wastyn (2009) believe that firms that use particular techniques would realize higher innovation performance with respect to product innovation and process innovation [44]. However, simple improvement in digital capabilities may not directly drive sustainable development of enterprises because, with increasing attention and enhancement of competitors in digital transformation, the advanced sustainable advantage achieved by an enterprise relying on digital technology is likely to be overtaken by latecomers. Therefore, especially for new ventures, it is necessary to pay attention to absorption, integration, and innovation of green knowledge to cope with the dynamic changes in the market’s demand for green transformation and governance and ensure that enterprises have a sustainable and stable competitive advantage in the face of latecomers.

Under the guidance of a sustainable development strategy, digital capabilities promote enterprises’ efforts to acquire green knowledge through green R&D cooperation, organization learning, and enhancing employee literacy, and they integrate discrete internal knowledge and discovered external knowledge by using the knowledge creation spiral, thus improving retention of that green knowledge. After socialization, externalization, combination, and internalization, the green knowledge creation process not only improves the green knowledge reserve of enterprises but also provides them a new business philosophy [32]. On the one hand, integration of digital capabilities into green innovation strategies enables enterprises to more efficiently transform green knowledge into assets with real value [39]. The resulting green knowledge benefits reduce marginal transaction costs and improve the market initiative of enterprises. On the other hand, digital capabilities make the green knowledge creation process more transparent, and, as the traceability of environmental performance, economic performance, and social performance is constantly improved, enterprises can identify and trace possible non-sustainable behaviors in the product chain [45]. This transformation trend has also greatly improved the autonomy of employees in making decisions by using big data information. The business model of digital technology helping employees to explore innovation opportunities helps strengthen managers’ sense of knowledge, and it promotes transformation of business knowledge into sustainable capabilities, thereby improving sustainable development performance. Based on the above analysis, this paper proposes the following hypothesis.

**Hypothesis** **3** **(H3).** *Green knowledge creation can play a mediating role between digital capability and sustainable development of new ventures*.

### 2.4. The Moderating Effect of Green Pressure

With evolution of new technologies and diversification of consumer demand, the competition of enterprises is no longer limited to the simple interests of those enterprises but also involves competition of multiple stakeholders, such as suppliers, enterprises, and clients. Due to the high cost and high uncertainty of achievement of sustainable development, enterprises are reluctant to independently carry out green innovation [46], and green collaborative cooperation among stakeholders helps to improve enthusiasm of enterprises to carry out green activities. For example, Yang and Lin (2020) believe that a common concern and participation of enterprises and suppliers in green activities can promote realization of energy conservation goals [47]. Jabbour et al. (2015) believe that enterprises will integrate the needs of stakeholders into their strategic formulations to seek resource support and will improve innovation through green cooperation in the market [48]. González-Moreno et al. (2019) proposed that collaborative cooperation and knowledge sharing are the main ways for enterprises to interact with suppliers and customers in the process of green innovation [49], which is reflected in the green consensus and green management cooperation reached between enterprises and their partners in the process of operation. It is also reflected in the actions of enterprises and partners in sharing green information and actively carrying out R&D cooperation. Therefore, sustainable development of enterprises cannot be separated from green pressure of stakeholders.

Green pressure reflects the impact of environmental awareness and behavior of suppliers and customers on enterprise innovation. At the level of high green pressure, green knowledge creation can often make enterprises actively adapt to the green development trend of the market, and acquisition and creation of new knowledge provide support for sustainable development of enterprises. Higher green pressure strengthens the driving effect of green knowledge creation for sustainable development. On the one hand, green pressure of suppliers reflects the impact of suppliers’ environmental awareness and environmental protection behaviors on green innovation of enterprises [50]. The stronger the suppliers’ environmental awareness, the more likely it is to affect resources selection of enterprises through the supply chain [51], thereby strengthening the positive impact of green knowledge creation on sustainable development. On the other hand, green pressure of customers reflects customers’ demands for green products or services [50]. The increase in green pressure from customers has reduced environmental uncertainty since it stimulates enterprises to seek green knowledge, thus enhancing their reputation and meeting customer needs but also improving the enterprises’ enthusiasm for sustainable development [52]. Therefore, the interaction between green pressure and green knowledge creation may become a catalyst for sustainable development of enterprises. Some studies have also put forward short-term obstacles caused by the environmental protection pressures of competitors for sustainable development of an enterprise in contrast to their stimulating role [53]. Different green pressures and the impact of green knowledge creation on sustainable development will produce different results. Compared to enterprises with higher green pressure, enterprises with lower green pressure have more difficulty in obtaining sustainable advantages through knowledge creation. Based on the above analysis, this paper proposes the following hypothesis.

**Hypothesis** **4** **(H4).** *Green pressure can positively moderate the relationship between green knowledge creation and sustainable development of new ventures*.

Combining H3 and H4, our study further proposes a moderated mediation model. Green knowledge creation plays a mediating role in the relationship between digital capabilities and sustainable development of new ventures, but the size of its role depends on the level of green pressure. When green pressure is high, willingness of new ventures to acquire green knowledge increases, and the digital capabilities effectively reduce the cost and risk of enterprises’ search and integration of green knowledge, improve the efficiency of green knowledge creation, and increase the advantage of sustainable development. When green pressure is low, new enterprises do not have high demand for green knowledge. Even though digital capabilities drive the creation process of green knowledge, lower green pressure reduces the effects of green knowledge creation, leading to an insignificant impact on sustainable development. Therefore, green pressure may have a positive impact on the path of ‘digital capability–green knowledge creation–sustainable development’. Based on the above analysis, this paper proposes the following hypothesis.

**Hypothesis** **5** **(H5).** *Green pressure can positively moderate the process of digital capability influencing sustainable development of new ventures through green knowledge creation*.

**Figure 1:**
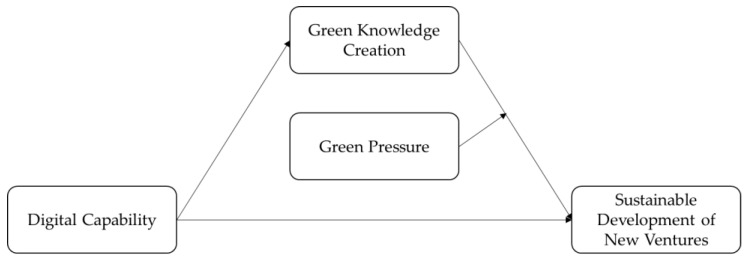
Theoretical structural model.

Integrating the above relationships and all the hypotheses, H1–H5, this study proposes the following theoretical structural model, as shown in Figure 1. Based on knowledge creation spiral theory, we believe that new ventures require rich knowledge resources to achieve sustainable development and that digital capability significantly increases the efficiency of knowledge creation, enabling enterprises to develop and share knowledge scattered in the network of stakeholders in the dynamic process of knowledge creation and under green pressure, thus stimulating new ventures to establish sustainable development advantages. Therefore, we propose assumptions that digital capability can promote sustainable development of new ventures, that green knowledge creation plays a mediating role in the impact mechanism of digital capability on sustainable development, and that green pressure can positively moderate the relationship between green knowledge creation and sustainable development. Furthermore, we believe that green pressure can positively moderate the mediating role of green knowledge creation.

## 3. Research Design

### 3.1. Data Sources

This paper used questionnaires to complete data collection and acquisition. Based on Song et al. (2008) [54], we chose new ventures in China that were more than one year old but less than eight years old as the survey targets. In order to ensure overall understanding of the survey content, we issued questionnaires to the founders, senior managers, and middle managers in charge of the business departments of the new ventures. Generally, middle and senior managers have a clearer understanding of an enterprise’s own digital layout and green knowledge innovation.

In order to avoid sample selection bias and endogenous problems, this study conducted data collection in stages by combining online and offline methods. Before the formal survey, the questionnaire was further improved through feasibility evaluation by experts in the field of digitalization and green innovation and also a pre-survey of 20 MBA students to ensure the content validity of the questionnaire. The sources of formal survey data were as follows: in the first stage, from May to June 2022, we contacted and issued questionnaires to the MBA and EMBA alumni groups of universities in Yangtze River Delta cities. In the second stage, in order to avoid errors caused by the ‘selection’ problem, provinces with different economic development levels and marketization were selected in which to carry out data collection. In August 2022, this study used the research group’s network to contact the management committees of science and technology entrepreneurship parks and university science and technology parks in Jiangsu, Zhejiang, Anhui, and Jiangxi Provinces to ask them to issue questionnaires or to provide us with lists of enterprises, and we then issued questionnaires directly to those enterprises. In summary, a total of 580 questionnaires were distributed in the two stages, 374 questionnaires were returned, and the questionnaire return rate was 64.48%. After eliminating the samples that did not meet the requirements for reasons such as being incomplete, having been filled out in an irregular way, or the enterprises not being new ventures, a total of 316 valid questionnaires remained, and the questionnaire effective return rate was 54.48%. The distribution characteristics of the sample enterprises are shown in Table 1.

This study then verified the reliability and validity of the questionnaires and tested the model’s correctness through the hierarchical regression method under the condition of including control variables. In order to further observe the relationship between variables, we used the three-step method to test the mediation mechanism of the model and used the bootstrap method to test the moderated mediation effect. At the same time, in order to ensure the robustness of the model and better understand the impact of digital capability and green knowledge creation on sustainable development, we used the path test based on bootstrap to again verify the mediating role of green knowledge creation.

### 3.2. Survey Instruments

The questionnaire was divided into five parts. The first part was for basic information about the enterprise, which can be considered as a control variable; the other four sub-scales are shown below. The scales were based on the mature scales of relevant variables, and some items were appropriately modified and adjusted according to the agility characteristics of new ventures and the actual research situation. A Likert five-point scale was adopted for all scales in this study, on which ‘1’ meant strongly disagree and ‘5’ meant strongly agree.

Digital capability: Measurement of this variable was mainly based on the research of Lenka et al. (2016) [55], which measured it using three aspects: intelligence capability, connection capability, and analytical capability. The scale included six items, such as ‘Is your enterprise able to enhance intelligent functionality through embedded smart components?’, ‘Do you think the enterprise has the ability to transmit signals and data to the cloud through wireless?’, ‘Do you think the enterprise can predict customer insight through logical data processing?’, etc.

Green knowledge creation: Measurement of this variable was mainly based on the research of Sabherwal and Becerra-Fernandez (2003) [56], which measured it using four aspects: socialization, externalization, combination, and internalization of green knowledge. The scale included 13 items, such as ‘Does the enterprise have a green problem-solving system based on a technology like case-based reasoning?’, ‘Has your enterprise brainstormed to solve problems in development through retreats or camps?’, ‘Does the enterprise bring employees into the knowledge field of the organization through learning-by-doing, training, and exercises?’, etc.

Green pressure: Measurement of this variable was mainly based on the research of Zhang et al. (2015) [57] and Xu J.Z. et al. (2017) [58], and it was measured for both suppliers and customers. The scale included eight items, such as ‘Most of the suppliers of the enterprise have high green requirements’, ‘Most of the suppliers of the enterprise have a high awareness of environmental protection’, etc.

Sustainable development: Sustainability depended on three fundamental pillars: economic, environmental, and social, and many studies have supported this view. Similarly, Elkington’s ‘triple bottom line’ principle also argued that enterprises should pay attention to the overall goals of economic prosperity, environmental protection, and social welfare while pursuing innovation performance [15]. Therefore, our research referred to the scale developed by Lee J. et al. to measure sustainable development of new ventures using the dimensions of economic, environmental, and social factors [59], and it included three items, such as ‘My enterprise provides technology, management, and financial assistance to solve social problems’, ‘We have implemented a quality and environmental management system such as ISO18000/14000′, etc.

### 3.3. Analysis Technique

In this study, SPSS 23.0 software (IBM, Armonk, NY, USA) was used for descriptive statistical analysis and correlation analysis, Mplus 8.0 software (Linda Muthén & Bengt Muthén, USA) was used for confirmatory factor analysis and regression analysis, and the process loader of SPSS was used to test the mediating effect and moderated mediation effect based on bootstrap method.

## 4. Empirical Analysis

### 4.1. Reliability and Validity Test

First, we calculated that the Cronbach’s α coefficient values for each latent variable were greater than 0.7, which is higher than the acceptable threshold value, indicating that the reliability of the scale was good. Second, the KMO values for each variable were greater than 0.8, and the Sig value for the Bartlett spherical test was 0, which meant that factor analysis could be performed. Therefore, we calculated CR and AVE to test the aggregate validity of the scale, in which AVE reflected the average explanatory ability of variables for items; according to Table 2, the minimum value of CR was 0.760, so more than 0.7, and the minimum value of AVE was 0.515, so more than 0.5, meaning both were higher than the threshold requirements, indicating that the aggregation validity of the model was acceptable. Finally, we tested the discriminant validity between the two latent variables. From Table 3, we can see that the fitting indexes of the M1 model all meet the fitness standard and were superior to other factor combinations, and, as shown in Table 4, the AVE square root values of each variable were greater than the correlation coefficient among all the latent variables, indicating that the latent variables had good discriminant validity.

### 4.2. Common Method Deviation Test and Collinearity Analysis

Since the sample data were collected using the same questionnaire, it was necessary to test the common method deviation. The study used CFA and ULMC methods to detect common method problems. If the CFA fitting index of the single factor model is the worst compared with the data of other factor combinations, it proves that the common method deviation is not serious. Table 3 shows that the fitness of the M5 model was the worst. In addition, according to the research of Podsakoff et al. [60], common method deviation is checked through controlling the unmeasured potential method factor, and a common method factor is added based on the M1 model such that the common method factor has the same load value on all items. Therefore, judged by the improvement degree of the fitting index, the fitting index of the four factors model + method factor was χ2/df = 2.440, CFI = 0.933, TLI = 0.922, RMSEA = 0.068, and SRMR = 0.056. Compared with the M1 model, the change amplitudes of CFI and TLI were 0.001 and 0, respectively, so less than 0.1, and the change amplitudes of RMSEA and SRMR were 0 and 0.011, respectively, so less than 0.05, indicating that the common method deviation of samples was not serious. The VIFs of all variables were below the recommended threshold of 5, indicating that the multi-collinearity problem did not affect the effectiveness of the model.

### 4.3. Correlation Analysis

Descriptive statistics and correlation analysis were conducted for all variables, as shown in Table 4, and there was a significant correlation between the core variables. From the correlation coefficient between variables, there was a significant positive correlation between digital capability and green knowledge creation (β = 0.596, *p* < 0.01); green knowledge creation was significantly positively correlated with sustainable development (β = 0.596, *p* < 0.01), and digital capability was significantly positively correlated with sustainable development (β = 0.458, *p* < 0.01). All the variables were significantly correlated, which provided preliminary support for the subsequent hypothesis testing process.

### 4.4. Hypothesis Test

In this study, the hierarchical regression method was used for hypothesis testing. Core explanatory variables were added to the benchmark model that only contained control variables. In order to exclude the heterogeneous impact of different industries and individual companies on sustainable development of new ventures, this study controlled the enterprise nature, size, age, and industries involved. The regression models and test results are shown in Table 5.

M4 showed the regression result of digital capability on sustainable development of new enterprises. Evidently, addition of digital capabilities improved the explanatory power of the model (ΔR^2^ = 0.207), and digital capabilities had a significant positive correlation with sustainable development (β = 0.468, *p* < 0.001). This showed that digital capability can effectively empower sustainable development of new enterprises. Therefore, hypothesis H1 was supported; that is, the main effect was significant.

Our research used the three-step method to verify the mediating role of green knowledge creation. First, M2 showed the regression result of digital capability on green knowledge creation; compared with M1, the explanatory power of M2 was significantly improved (ΔR^2^ = 0.339), and there was a significant positive correlation between digital ability and green knowledge creation (β = 0.598, *p* < 0.001). Hypothesis H2 was supported, which proved that the technical effect of digital capabilities accelerated integration of enterprise knowledge, thus providing a solid available resource base for green knowledge creation. Second, M5 showed the regression result of green knowledge creation on sustainable development; compared with M1, the explanatory power of M5 was significantly improved (ΔR^2^ = 0.362), and there was a significant positive correlation between green knowledge creation and sustainable development (β = 0.610, *p* < 0.001). This proved that the socialization, externalization, combination, and internalization processes of green knowledge can accumulate innovation advantages while strengthening knowledge creation ability of new ventures, further stimulating their sustainable development potential. Finally, we tested the mediating role of green knowledge creation. In M6, digital capability and green knowledge creation were taken as independent variables and sustainable development was taken as the dependent variable for regression. The regression of digital ability on sustainable development was still significant (β = 0.159, *p* < 0.01), but the regression coefficient decreased from 0.468 to 0.159. The regression of green knowledge creation was also significant, indicating that the impact of digital capability on sustainable development is weakened due to the addition of green knowledge creation. Therefore, hypothesis H3 was supported; that is, the mediating effect was significant.

In order to test the moderating role of green pressure between green knowledge creation and sustainable development of new enterprises, this study took sustainable development as a dependent variable, successively added control variables, the mediating variable (green knowledge creation), and the moderating variable (green pressure), and finally added the interaction item of mediating variable and moderating variable. In order to avoid the collinearity problem, green knowledge creation and green pressure were centralized. As shown in M8, the interaction between green knowledge creation and green pressure had a significant positive impact on sustainable development (β = 0.668, *p* < 0.001), indicating that, the higher the green pressure, the more conducive green knowledge creation activities of new enterprises are to their sustainable development. Therefore, hypothesis H4 was supported; that is, the moderating effect was significant.

**Figure 2:**
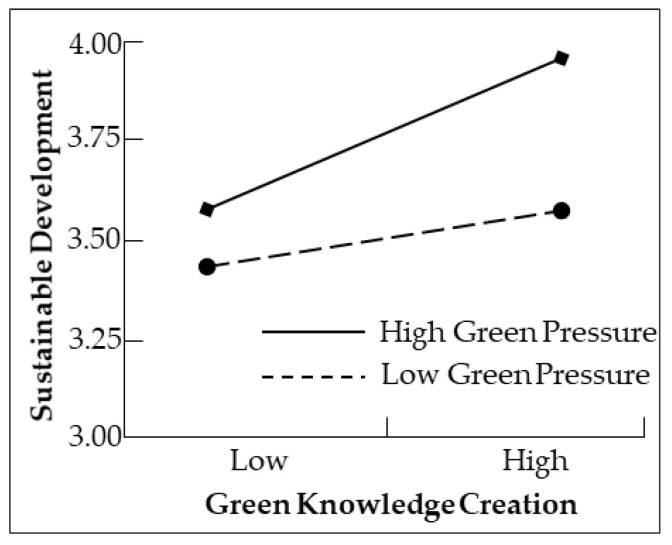
Slope figure of green pressure moderating effect.

In order to clearly display the moderation relationship, according to the simple slope analysis proposed by Aiken and West (1991) [61], based on green pressure, there is a standard deviation of plus or minus, and its moderating role is shown in Figure 2. The high green pressure made the positive effect of green knowledge creation of new ventures on sustainable development stronger; on the contrary, low green pressure made the positive effect of green knowledge creation on sustainable development weaker, which verified the hypothesis of moderating effect.

Furthermore, we tested the moderated mediation effect. In this study, Mplus was used to perform 5000 iterations of the bootstrap method to test the moderated mediation effect; the results are shown in Table 6. Based on green pressure’s mean value + 1SD, the confidence interval at the 95% level of the mediation path ‘digital capability–green knowledge creation–sustainable development’ did not contain 0, indicating that, when green pressure was high, green pressure played a significant role in moderating the process of digital capability influencing sustainable development through green knowledge creation. Based on green pressure’s mean value—1SD—or when the green pressure was low, the confidence interval of this mediation path at the 95% level was [0.142, 0.351], which also did not contain 0 and had a significant moderating effect. Therefore, the greater the green pressure, the more positive the impact of digital capability on sustainable development through green knowledge creation. The moderated mediation effect value was 0.053 and the 95% confidence interval was [0.014, 0.125], excluding 0, which also proved that green pressure had a significant positive moderating effect on the mediation path of ‘digital capability–green knowledge creation–sustainable development’ overall. Therefore, hypothesis H5 was supported; that is, the moderated mediation effect was significant.

### 4.5. Robustness Test

In order to test the reliability and robustness of the above conclusions, based on the test of mediating effect, the bootstrap method with deviation correction was used to further test, extract 5000 times repeatedly, and verify the significance of the mediation path under a 95% confidence interval. The results are shown in Table 7. The 95% confidence interval of the bootstrap of the mediation path ‘digital capability–green knowledge creation–sustainable development’ was [0.197, 0.375], excluding 0, so the mediating effect was significant. At the same time, the direct effect of digital capability on sustainable development was 0.140, the 95% confidence interval of bootstrap was [0.038, 0.242], the total effect was 0.420, and the confidence interval was [0.329, 0.511]. Both the direct effect and the total effect were significant. The results of the robustness analysis further supported the hypothesis of this study.

## 5. Conclusions and Discussion

### 5.1. Research Conclusions

This paper investigated the impact of digital capability on sustainable development of new ventures under the dual impact of green knowledge creation and green pressure using digitalization as the entry point. The empirical results show that, first, digital capability has a positive impact on sustainable development of new ventures. This result is similar to the research of Zhou Q. and Wang S. (2021) [31], and it shows that new ventures can deepen their competitive advantages through improving their digital capability, which is conducive to improved performance in terms of environmental, economic, and social benefits and promotes sustainability. Second, green knowledge creation plays a part in the mediating role between digital capability and sustainable development. Through effective digital construction and application, new ventures can quickly integrate, create, and spread green knowledge based on the opportunity of pre-development, thereby helping the enterprises shape sustainability. Third, green pressure positively moderates the relationship between green knowledge creation and sustainable development. Higher green pressure can stimulate the efficiency of green knowledge creation of enterprises, strengthen the output of that green knowledge, and improve the stability of sustainable development of enterprises. Fourth, green pressure positively moderates the process of digital capability influencing sustainable development through green knowledge creation. Under high green pressure, the demand of new ventures for green knowledge increases, and having digital capability effectively reduces the cost and risk of enterprises’ search for and integration of green knowledge, improves the efficiency of green knowledge creation, and increases the sustainability of green development.

### 5.2. Managerial Implications

Based on the above conclusions, the following three implications for the management practice of enterprises are summarized.

First, in the digital economy era, new ventures need to establish effective sustainable development advantages. In the Action Plan for Innovative Development of the Industrial Internet (2021–2023), the Industrial Internet Task Force of China proposed to build a digital platform, system solutions, products, and services that meet the needs of new enterprises to promote their digital capability and share orders, production, and resources. Evidently, digital transformation has become an inevitable theme for contemporary enterprises, and new enterprises need to follow the trend of the digital economy, constantly build digital capability, exploit potential opportunities in the market by using digital traceability and data mining technology, establish business insights in the process of learning, integration, and creation by using cloud platforms, and fully transform their own capability into sustainable products and services.

Second, the process of green knowledge creation is crucial for sustainable development of new ventures through digital capability. On the one hand, new green knowledge does not only accelerate the learning process of enterprises but also aids in implementing the concept of green development into the production, operation, marketing, and services of the organization, thus aiding enterprises in achieving sustainable development driven by digital capability. On the other hand, new ventures can create and share green knowledge through dynamic conversion between explicit knowledge and tacit knowledge. Therefore, new ventures should use digital means to reasonably allocate internal and external knowledge resources, establish green knowledge barriers, and improve business performance and competitiveness of enterprises.

Third, in the process of enterprises creating green knowledge and transforming it into sustainable competitive advantage, green pressure from stakeholders has played a positive role. Higher green pressure can improve the impact of green knowledge creation. Nowadays, new ventures need to select suppliers with green supply capability and purchase raw materials that meet environmental protection standards from suppliers; at the same time, it is also necessary to improve their adaptability to green pressure by launching products and services with green and sustainable characteristics under green pressure of customers.

### 5.3. Limitations and Future Research Directions

This study explored the process by which new ventures use digital capability to achieve sustainable development from the perspective of knowledge creation, and it provided a new perspective for the study of digitalization. However, this study has some shortcomings.

First, since the data used in this study were cross-sectional, it lacked a view of the dynamic impact of digital capability and green knowledge creation over time. Therefore, in future research, data should be collected from different periods to show how digital capability and green knowledge creation affect sustainable development of new ventures over time.

Second, the number of survey samples involved in this study was limited, and the range of industries involved was not comprehensive. Perhaps, due to the particularity of the research group, the results of this study cannot represent the situation of all new enterprises, so the research results need to be verified with a larger sample size.

Finally, this study only discussed the contingency impact of green pressure in the process of knowledge creation, but the knowledge creation process for enterprises is often affected by organizational inertia, organizational resilience, and other internal characteristics. Therefore, future research can be conducted considering these perspectives to further enrich the research model.

## Figures and Tables

**Table 1:** Distribution of sample enterprises.

Survey Item	Classification	Number	Ratio (%)
Enterprise Nature	State-owned	45	14.24%
	Private-owned	148	46.84%
	Foreign-owned	37	11.71%
	Other	86	27.22%
Enterprise Age	1–3	129	40.82%
	4–6	91	28.80%
	7–8	96	30.38%
Staff Size	≤20	22	6.96%
	21–50	31	9.81%
	51–100	79	25.00%
	101–200	107	33.86%
	>200	77	24.37%
Industry Involved	Biomedicine	19	6.01%
	Information Software	68	21.52%
	New Energy	55	17.41%
	New Material	27	8.54%
	High-end Equipment Manufacturing	92	29.11%
	Energy Saving and Environmental Protection	21	6.65%
	Other	34	10.76%

**Table 2:** Reliability and aggregation validity test.

Variable	Item	Cronbach’s α	AVE	CR	KMO
Digital Capability	DC1 Enterprises enhance intelligent functions through embedded smart components	0.919	0.564	0.920	0.928
	DC2 Enterprises use and operate data to sense and capture				
	DC3 Enterprises transmit signals and data to the cloud wirelessly				
	DC4 Enterprises realize networking functions through inter-connected assets				
	DC5 Enterprises predict customer insights through logical data processing				
	DC6 Enterprises realize value visualization through simulation of scenarios				
Green Knowledge Creation	GKC1 Enterprises have green problem-solving system based on a technology, such as case-based reasoning	0.884	0.607	0.885	0.874
	GKC2 Enterprises have groupware and other team collaboration tools				
	GKC3 There is green content in the enterprise’s professional knowledge guide				
	GKC4 Enterprises can perform green modeling based on analogies and metaphors				
	GKC5 Enterprises include green-related indicators into the database				
	GKC6 Enterprises reflect green elements in the webpage				
	GKC7 Enterprises have cross-directorate green cooperation projects				
	GKC8 Enterprises use apprentices and mentors to transfer green knowledge				
	GKC9 Enterprises solve green problems through retreats or camps				
	GKC10 Enterprises have a cross-regional staff rotation system				
	GKC11 Enterprises have special employee green training				
	GKC12 Enterprises encourage employees to learn through doing				
	GKC13 Enterprises encourage employees to learn through observation				
Green Pressure	GP1 Most suppliers have high green requirements	0.816	0.610	0.823	0.810
	GP2 Most suppliers use green innovation as an important indicator of reputation of enterprises				
	GP3 Most suppliers are willing to provide environmental protection materials				
	GP4 Most suppliers have high environmental awareness				
	GP5 Most customers have high demand for green products				
	GP6 Most customers are very concerned about green innovation behavior of enterprises				
	GP7 Customers require products to meet environmental standards				
	GP8 Customers value products with green concept				
Sustainable Development	SD1 The enterprise has implemented environmental management and evaluation system (ISO 180000/14000)	0.793	0.515	0.760	0.819
	SD2 Enterprises provide environmentally friendly products and services				
	SD3 Enterprises provide technical, managerial, or financial assistance to solve social problems				

**Table 3:** Results of confirmatory factor analysis.

Model Type	χ^2^	df	χ^2^/df	CFI	TLI	RMSEA	SRMR
M1: DC, GKC, GP, SD	402.046	164	2.452	0.932	0.922	0.068	0.045
M2: DC + GKC, GP, SD	828.064	167	4.958	0.812	0.786	0.112	0.075
M3: DC, GKC + GP, SD	739.902	167	4.431	0.837	0.815	0.104	0.082
M4: DC + GKC + GP, SD	1159.322	169	6.860	0.718	0.683	0.136	0.100
M5: DC + GKC + GP + SD	1248.365	170	7.343	0.693	0.657	0.142	0.102

Note: N = 316, DC = digital capability, GKC = green knowledge creation, GP = green pressure, SD = sustainable development.

**Table 4:** Descriptive statistics and correlation analysis.

Variable	1	2	3	4
1. Digital Capability	0.751			
2. Green Knowledge Creation	0.606 **	0.779		
3. Green Pressure	0.176 **	0.125 *	0.781	
4. Sustainable Development	0.458 **	0.596 **	0.102 *	0.718
Mean	3.853	3.899	3.603	3.642
Standard Deviation	0.678	0.636	0.746	0.623

Note: * means *p* < 0.05, ** means *p* < 0.01; diagonal values in the table are the square root of the AVE of the corresponding variable.

**Table 5:** Regression analysis results.

Variable	Green Knowledge Creation		Sustainable Development					
	M1	M2	M3	M4	M5	M6	M7	M8
**Control variables**								
Enterprise Nature	0.136	0.056	−0.023	−0.086	−0.106	−0.115 *	−0.108	−0.110 *
Enterprise Age	0.039	0.003	0.028	0.001	0.005	−0.001	0.003	0.006
Staff Size	0.106	−0.002	0.131 *	0.047	0.067	0.048	0.067	0.064
Industry Involved	−0.169 *	−0.074	0.081	0.156 *	0.184 **	0.194 **	0.187 **	0.183 **
**Independent variable**								
Digital Capability		0.598 ***		0.468 ***		0.159 **		
**Mediator**								
Green Knowledge Creation					0.610 ***	0.517 ***	0.608 ***	0.596 ***
Moderator								
Green Pressure							0.223 **	0.117 *
**Interaction**								
Green Knowledge Creation × Green Pressure								0.668 ***
R^2^	0.035	0.371	0.023	0.230	0.383	0.398	0.383	0.398
Adj_R^2^	0.022	0.361	0.011	0.218	0.373	0.387	0.371	0.374
F	2.772 *	36.516 ***	1.835	18.467 ***	38.327 ***	33.987 ***	31.905 ***	37.771 ***

Note: N = 316; * means *p* < 0.05, ** means *p* < 0.01, *** means *p* < 0.001.

**Table 6:** Bootstrap-moderated mediation path verification.

Indirect Impact Path	Moderator	Estimate	Boot SE	95% Confidence Interval
Mediation Effect	Low Green Pressure	0.237	0.053	[0.142, 0.351]
	Middle Green Pressure	0.276	0.046	[0.191, 0.372]
	High Green Pressure	0.316	0.052	[0.217, 0.428]
Moderated Mediation Effect	—	0.053	0.035	[0.014, 0.125]

**Table 7:** Bootstrap mediation path verification.

Paths	Effect	Estimate	Boot SE	95% Confidence Interval	Proportion
Path1: Digital Capability → Sustainable Development	Direct Effect	0.140	0.052	[0.038, 0.242]	33.3%
Path2: Digital Capability → Green Knowledge Creation → Sustainable Development	Mediating Effect	0.280	0.046	[0.197, 0.375]	66.7%
Path1 + Path2	Total Effect	0.420	0.046	[0.329, 0.511]	100%

## Data Availability

Not applicable.

## References

[1] Carroll A.B., Buchholtz A.K. (2014). Business and Society: Ethics, Sustainability, and Stakeholder Management.

[2] Elia G., Margherita A., Passiante G. (2020). Digital entrepreneurship ecosystem: How digital technologies and collective intelligence are reshaping the entrepreneurial process. Technol. Forecast. Soc. Chang..

[3] Rachinger M., Rauter R., Müller C., Vorraber W., Schirgi E. (2019). Digitalization and its influence on business model innovation. J. Manuf. Technol. Manag..

[4] Vial G. (2019). Understanding digital transformation: A review and a research agenda. J. Strateg. Inf. Syst..

[5] Nambisan S., Lyytinen K., Majchrzak A., Song M. (2017). Digital Innovation Management: Reinventing innovation management research in a digital world. MIS Q..

[6] Khin S., Ho T.C. (2019). Digital technology, digital capability and organizational performance: A mediating role of digital innovation. Int. J. Innov. Sci..

[7] Ansong E., Boateng R. (2019). Surviving in the digital era–business models of digital enterprises in a developing economy. Digit. Policy Regul. Gov..

[8] Bag S., Pretorius J.H.C., Gupta S., Dwivedi Y.K. (2020). Role of institutional pressures and resources in the adoption of big data analytics powered artificial intelligence, sustainable manufacturing practices and circular economy capabilities. Technol. Forecast. Soc. Chang..

[9] Liu Z., Yao Y.X., Zhang G.S., Kuang H.S. (2020). Firm’s Digitalization, Specific Knowledge and Organizational Empowerment. China Ind. Econ..

[10] Jedynak M., Czakon W., Kuźniarska A., Mania K. (2019). Digital transformation of organizations: What do we know and where to go next?. J. Organ. Change. Manag..

[11] Correani A., De Massis A., Frattini F., Petruzzelli A.M., Natalicchio A. (2020). Implementing a digital strategy: Learning from the experience of three digital transformation projects. Calif. Manag. Rev..

[12] Nonaka I., Takeuchi H. (1996). The knowledge-creating company: How Japanese companies create the dynamics of innovation. Long Range Plan..

[13] Lee J.W., Kim Y.M., Kim Y.E. (2018). Antecedents of adopting corporate environmental responsibility and green practices. J. Bus. Ethics.

[14] Kawai N., Strange R., Zucchella A. (2018). Stakeholder pressures, EMS implementation, and green innovation in MNC overseas subsidiaries. Int. Bus. Rev..

[15] Elkington J. (1998). Partnerships from cannibals with forks: The triple bottom line of 21st-century business. Environ. Qual. Manag..

[16] Hall J., Vredenburg H. (2003). The challenge of innovating for sustainable development. MIT Sloan Manag. Rev..

[17] Magnusson T., Lindström G., Berggren C. (2003). Architectural or modular innovation? Managing discontinuous product development in response to challenging environmental performance targets. Int. J. Innov. Manag..

[18] Cohen W.M., Levinthal D.A. (1990). Absorptive capacity: A new perspective on learning and innovation. Adm. Sci. Q..

[19] Du L., Zhang Z., Feng T. (2018). Linking green customer and supplier integration with green innovation performance: The role of internal integration. Bus. Strategy Environ..

[20] Xiao Y., Dang X.H., Xiang X.Y. (2017). A research on the innovation network governance based on cultural heterogeneity. Sci. Res. Manag..

[21] Hockerts K., Wüstenhagen R. (2010). Greening Goliaths versus emerging Davids—Theorizing about the role of incumbents and new entrants in sustainable entrepreneurship. J. Bus. Ventur..

[22] Divito L., Bohnsack R. (2017). Entrepreneurial orientation and its effect on sustainability decision tradeoffs: The case of sustainable fashion firms. J. Bus. Ventur..

[23] Chen Y., Shi J.G., Zhang H. (2021). Review and prospects of sustainable entrepreneurship research. Stud. Sci. Sci..

[24] International Energy Agency Digitalization and Energy. https://iea.blob.core.windows.net/assets/b1e6600c-4e40-4d9c-809d-1d1724c763d5/DigitalizationandEnergy3.pdf.

[25] Mondejar M.E., Avtar R., Diaz H.L.B., Dubey R.K., Esteban J., Gómez-Morales A., Hallam B., Mbungu N.T., Okolo C.C., Prasad K.A. (2021). Digitalization to achieve sustainable development goals: Steps towards a Smart Green Planet. Sci. Total Environ..

[26] Appio F.P., Frattini F., Petruzzelli A.M., Neirotti P. (2021). Digital transformation and innovation management: A synthesis of existing research and an agenda for future studies. J. Prod. Innov. Manag..

[27] Hagstrom M. (2012). High-performance analytics fuels innovation and inclusive growth: Use big data, hyperconnectivity and speed to intelligence to get true value in the digital economy. J. Adv. Anal..

[28] Kunkel S., Matthess M. (2020). Digital transformation and environmental sustainability in industry: Putting expectations in Asian and African policies into perspective. Environ. Sci. Policy.

[29] Smart Heating Maximizes Efficiency with Precision Control. https://www.huaweicloud.com/intl/en-us/cases/tpgr.html.

[30] Zhou Q., Wang S. (2021). Study on the relations of supply chain digitization, flexibility and sustainable development—A moderated multiple mediation model. Sustainability.

[31] Habanik J., Grencikova A., Krajco K. (2019). The impact of new technology on sustainable development. Eng. Econ..

[32] Nonaka I. (1994). A dynamic theory of organizational knowledge creation. Organ. Sci..

[33] Miller D. (2011). Miller (1983) revisited: A reflection on EO research and some suggestions for the future. Entrep. Theory Pract..

[34] Lin Y.H., Chen Y.S. (2017). Determinants of green competitive advantage: The roles of green knowledge sharing, green dynamic capabilities, and green service innovation. Qual. Quant..

[35] Zhang X.E., Huang H.S., Xu X.J., Xue M. (2021). Research on the influence of green entrepreneurial orientation on green competitive advantage. Stud. Sci. Sci..

[36] Tu X.Y., Yan X.L. (2022). Digital transformation, knowledge spillover, and enterprise total factor productivity: Empirical evidence from listed manufacturing companies. Ind. Econ. Res..

[37] Morton J., Wilson A.D., Cooke L. (2020). The digital work of strategists: Using open strategy for organizational transformation. J. Strateg. Inf. Syst..

[38] Li R., Rao J., Wan L. (2022). The digital economy, enterprise digital transformation, and enterprise innovation. Manag. Decis. Econ..

[39] Wu A., Li T. (2020). Gaining sustainable development by green supply chain innovation: Perspectives of specific investments and stakeholder engagement. Bus. Strategy Environ..

[40] Lin K.Y. (2018). User experience-based product design for smart production to empower industry 4.0 in the glass recycling circular economy. Comput. Ind. Eng..

[41] Wood M.S., Mckinley W. (2017). After the venture: The reproduction and destruction of entrepreneurial opportunity. Strateg. Entrep. J..

[42] Wang M.Y., Li Y.M. (2021). Equilibrium and stability of green technology innovation system with multi-agent participation. Chin. J. Manag. Sci..

[43] Chen J., Huang S., Liu Y.H. (2020). Operations management in the digitization era: From empowering to enabling. J. Manag. World.

[44] Czarnitzki D., Wastyn A. (2009). Does Professional Knowledge Management Improve Innovation Performance at the Firm Level?.

[45] Cousins P.D., Lawson B., Petersen K.J., Fugate B. (2019). Investigating green supply chain management practices and performance: The moderating roles of supply chain ecocentricity and traceability. Int. J. Oper. Prod. Manag..

[46] Ma W., Zhang R., Chai S. (2019). What drives green innovation? A game theoretic analysis of government subsidy and cooperation contract. Sustainability.

[47] Yang Z., Lin Y. (2020). The effects of supply chain collaboration on green innovation performance: An interpretive structural modeling analysis. Sustain. Prod. Consum..

[48] Jabbour C.J.C., Neto A.S., Gobbo J.A., de Souza Ribeiro M., de Sousa Jabbour A.B.L. (2015). Eco-innovations in more sustainable supply chains for a low-carbon economy: A multiple case study of human critical success factors in Brazilian leading companies. Int. J. Prod. Econ..

[49] González-Moreno Á., Triguero Á., Sáez-Martínez F.J. (2019). Many or trusted partners for eco-innovation? The influence of breadth and depth of firms’ knowledge network in the food sector. Technol. Forecast. Soc. Chang..

[50] Hou Y.H., Li S.S., Hao M., Rao W.Z. (2021). Influence of market green pressure on the green innovation behavior of knowledge-based enterprises. China Popul. Resour. Environ..

[51] Cao H.J., Chen Z.W. (2017). The driving effect of internal and external environment on green innovation strategy: The moderating role of top management’s environmental awareness. Nankai Bus. Rev..

[52] Zhang B., Bi J., Yuan Z., Ge J., Liu B., Bu M. (2008). Why do firms engage in environmental management? An empirical study in China. J. Clean. Prod..

[53] Wu C.Y., Wu D. (2009). Research on the forming path of market-oriented enterprise green management behaviors. Nankai Business Review.

[54] Song M., Podoynitsyna K., Van Der Bij H., Halman J.I. (2008). Success factors in new ventures: A meta-analysis. J. Prod. Innov. Manag..

[55] Lenka S., Parida V., Wincent J. (2017). Digitalization capabilities as enablers of value co-creation in servitizing firms. Psychol. Mark..

[56] Sabherwal R., Becerra-Fernandez I. (2003). An empirical study of the effect of knowledge management processes at individual, group, and organizational levels. Decis. Sci..

[57] Zhang B., Wang Z., Lai K.H. (2015). Mediating effect of managers’ environmental concern: Bridge between external pressures and firms’ practices of energy conservation in China. J. Environ. Psychol..

[58] Xu J.Z., Guan J., Lin Y. (2017). Institutional pressures, top managers’ environmental awareness and environmental innovation practices: An institutional theory and upper echelons theory perspective. Manag. Rev..

[59] Lee J.S., Kim S.K., Lee S.Y. (2016). Sustainable supply chain capabilities: Accumulation, strategic types and performance. Sustainability.

[60] Podsakoff P.M., MacKenzie S.B., Lee J.Y., Podsakoff N.P. (2003). Common method biases in behavioral research: A critical review of the literature and recommended remedies. J. Appl. Psychol..

[61] Aiken L.S., West S.G., Reno R.R. (1991). Multiple Regression: Testing and Interpreting Interactions.

